# Distribution and relative expression of vasoactive receptors on arteries

**DOI:** 10.1038/s41598-020-72352-5

**Published:** 2020-09-21

**Authors:** Xinhao Liu, Dan Luo, Jie Zhang, Lei Du

**Affiliations:** 1 Department of Anesthesiology and Translational Neuroscience Center, West China Hospital, Sichuan University, 37 Guoxuexiang, Chengdu, 610041 Sichuan China; 2 Key Laboratory of Transplant Engineering and Immunology, West China Hospital, Sichuan University, 37 Guoxuexiang, Chengdu, 610041 Sichuan China

**Keywords:** Cardiovascular biology, Medical research

## Abstract

Arterial tone is regulated by multiple ligand-receptor interactions, and its dysregulation is involved in ischemic conditions such as acute coronary spasm or syndrome. Understanding the distribution of vasoactive receptors on different arteries may help guide the development of tissue-specific vasoactive treatments against arterial dysfunction. Tissues were harvested from coronary, mesenteric, pulmonary, renal and peripheral human artery (n = 6 samples of each) and examined using a human antibody array to determine the expression of 29 vasoactive receptors and 3 endothelin ligands. Across all types of arteries, outer diameter ranged from 2.24 ± 0.63 to 3.65 ± 0.40 mm, and AVPR1A was the most abundant receptor. The expression level of AVPR1A in pulmonary artery was similar to that in renal artery, 2.2 times that in mesenteric artery, 1.9 times that in peripheral artery, and 2.2 times that in coronary artery. Endothelin-1 was expressed at significantly higher levels in pulmonary artery than peripheral artery (8.8 times), mesenteric artery (5.3 times), renal artery (7.9 times), and coronary artery (2.4 times). Expression of ADRA2B was significantly higher in coronary artery than peripheral artery. Immunohistochemistry revealed abundant ADRA2B in coronary artery, especially vessels with diameters below 50 μm, but not in myocardium. ADRA2C, in contrast, was expressed in both myocardium and blood vessels. The high expression of ADRA2B in coronary artery but not myocardium highlights the need to further characterize its function. Our results help establish the distribution and relative levels of tone-related receptors in different types of arteries, which may guide artery-specific treatments.

## Introduction

Arterial tone (extent of constriction) modulates the perfusion of vital organs and is well regulated by interactions between neurohumoral ligands and their receptors on vascular smooth muscle or endothelial cells^1,2^. Disorder of arterial tone may impair blood supply and lead to acute, potentially life-threatening organ dysfunction, such as coronary artery spasm^3^ or acute coronary syndrome^4^. A powerful treatment for such dysfunction would be vasodilators that act locally at the ischemic organ without affecting other vasculature, thereby maintaining pressure to push more blood to the target organ. Developing such specific vasodilators requires understanding the distribution of vasoactive receptors and their ligands across different artery types. For example, the cardiovascular system, especially the coronary artery, shows extensive sympathetic innervation and may be associated with cardiovascular diseases^6,7^. Investigating the expression pattern of various subtypes of adrenoceptors may help in treating ischemic heart disease. Endothelin-1 and its receptors are positively associated with cardiovascular diseases^8^, pulmonary arterial hypertension^9^, resistant hypertension^10,11^ and chronic kidney diseases^12^. Investigation of endothelin expression in different arteries may help develop treatments for different target organs.

As a step in this direction, the present study investigated the distribution of 29 types of receptors and 3 types of endothelin ligands related to vascular smooth muscle cells and endothelial cells in small and medium-sized arteries, which are believed to be the primary determinants of organ perfusion^13^. In particular, we focused on adrenoceptors in coronary arteries because the sympathetic nerve is involved in the pathophysiology of ischemic heart disease^14^, a leading cause of death worldwide^15^.

## Methods

### Tissue samples

Specimens of arteries were taken from patients 18–80 years old at West China Medical Center of Sichuan University who underwent pulmonary lobotomy; resection of the kidney, intestine or muscle; or heart transplantation. Samples were excluded from the study if the patients had systemic vasculitis or any condition that might compromise ability to regulate vascular tone, including history of smoking, daily alcohol use, diabetes mellitus, or long-term use of corticosteroids.

Patients or family members provided written informed consent for their tissues to be taken and used for biomedical research. This study was approved by the Ethics Committee of West China Hospital and registered in the Chinese Clinical Trial Registry (https://www.chictr.org.cn/showprojen.aspx?proj=10753) as ChiCTR-OPC-15006229.

All samples were processed within 2 h after collection. Connective tissue surrounding the blood vessels was removed on ice, and samples were stored at − 80 °C until further analyses, for which they were thawed only once.

### Human antibody array

A portion of all artery samples was homogenized for 30 min with a high-speed dispersion cutter in cell lysis buffer containing protease inhibitor cocktail. Homogenates were centrifuged for 20 min at 4 °C, and the supernatant was processed using a membrane-based array of biotinylated human antibodies (catalog no. AAH-BLG-CUST, RayBiotech, Guangzhou, China) according to the manufacturer’s instructions. This kit detects three subtypes of endothelin and 29 receptors of seven types: nine adreno, nine cholinergic, two angiotensin, three vasopressin, four 5-hydroxytryptamine, one prostaglandin I2 and one thromboxane A2 receptor (Supplementary Fig. 1). The abbreviations of all detected ligands and receptors are listed in Table 1.

**Table 1:** Vasoactive receptors and ligands assayed in this study.

Abbreviation	Full name	GenBank ID
ADRA1A	Alpha1A-adrenoceptor	148
ADRA1B	Alpha1B-adrenoceptor	147
ADRA1D	Alpha1D-adrenoceptor	146
ADRA2A	Alpha2A-adrenoceptor	150
ADRA2B	Alpha2B-adrenoceptor	151
ADRA2C	Alpha2C-adrenoceptor	152
ADRB1	Beta1-adrenoceptor	153
ADRB2	Beta2-adrenoceptor	154
ADRB3	Beta3-adrenoceptor	155
AGTR1	Angiotensin II receptor 1	185
AGTR2	Angiotensin II receptor 2	186
AVPR1A	Arginine vasopressin receptor 1A	552
AVPR1B	Arginine vasopressin receptor 1B	553
AVPR2	Arginine vasopressin receptor 2	554
CHRM1	Cholinergic receptor muscarinic 1	1128
CHRM2	Cholinergic receptor muscarinic 2	1129
CHRM3	Cholinergic receptor muscarinic 3	1131
CHRM5	Cholinergic receptor muscarinic 5	1133
CHRNA1	Cholinergic receptor nicotinic alpha 1 subunit	1134
CHRNA2	Cholinergic receptor nicotinic alpha 2 subunit	1135
CHRNA3	Cholinergic receptor nicotinic alpha 3 subunit	1136
CHRNA4	Cholinergic receptor nicotinic alpha 4 subunit	1137
CHRNA5	Cholinergic receptor nicotinic alpha 5 subunit	1138
ET1	Endothelin 1	1906
ET2	Endothelin 2	1907
ET3	Endothelin 3	1908
HTR1A	5-hydroxytryptamine receptor 1A	3350
HTR1B	5-hydroxytryptamine receptor 1B	3351
HTR2A	5-hydroxytryptamine receptor 2A	3356
HTR2B	5-hydroxytryptamine receptor 2B	3357
PTGIR	Prostaglandin I2 receptor	5739
TBXA2R	Thromboxane A2 receptor	6915

Information was from GenBank.

Samples were processed according to the manufacturer’s instructions. After dialysis of samples, total protein concentration was determined, and primary amines were biotinylated at room temperature for 30 min. The sample was incubated in blocking buffer to remove unbound biotin, loaded onto the membrane array, covered with a lid to prevent drying out, and incubated at 4 °C overnight. The membrane was washed four times, incubated with horseradish peroxidase-conjugated streptavidin, then incubated in detection buffer. Signal on the membrane was detected using a chemiluminescence imaging analysis system (ImageQuant LAS4000 Scanner, GE Healthcare, USA), and signal intensity was quantified by densitometry. The array contained positive control spots of different concentrations of biotinylated bovine serum albumin (Pos1a, Pos2a, Pos3a and Pos1b, Pos2b, Pos3b), which served as reference points for orienting the arrays and normalizing results from different arrays.

### Immunohistochemistry of coronary artery

The posterior wall of the left ventricle was harvested, fixed with 4% paraformaldehyde, embedded in paraffin, and sliced into 6-μm sections on slides. Slices were deparaffinized, rehydrated, incubated with methanol containing 3% (v/v) H_2_O_2_ for 30 min to inactivate endogenous peroxidases, and heated in EDTA buffer in a 95 °C water bath for 45 min for antigen retrieval. Slides were then blocked with 5% bovine serum albumin for 1 h and incubated overnight at 4 °C with primary antibodies against the following receptors: ADRA1A (catalog no. ab137123, Abcam, Cambridge, UK), ADRA1B (ab169523, Abcam), ADRA1D (TA328712, Origene, Rockville, MD USA), ADRA2A (ab85570, Abcam), ADRA2B (AP17872PU-N, Origene), and ADRA2C (ab151618, Abcam). Slides were washed with phosphate-buffered saline and incubated for 1 h at 37 °C with goat anti-rabbit secondary antibodies (Jackson Immuno Research Laboratories, West Grove, PA, USA). Finally, the slides were developed with DAB chromogen (Thermo Fisher Scientific, Waltham, MA, USA) and counterstained with hematoxylin.

### Western blotting of all artery types

A portion of all artery samples was initially pulverized in liquid nitrogen, lysed in RIPA buffer containing protease inhibitor cocktail (Roche) for 1 h at 4 °C, and centrifuged at 12,000 g for 15 min at 4 °C. The supernatant was transferred to a fresh centrifuge tube, protein concentration was determined, and 30 μg of protein was separated by SDS-PAGE in 12% acrylamide gels and transferred onto nitrocellulose membranes. Membranes were blocked with 5% nonfat dry milk for 1 h at room temperature and incubated overnight at 4 °C with primary antibodies against ADRA2B (1:2000; AP17872PU-N, Origene) or GAPDH (1:5000; ab8245, Abcam). Membranes were washed in phosphate-buffered saline three times and incubated with goat anti-rabbit secondary antibodies for 1 h at room temperature. Finally, membranes were covered with ECL reagent (Thermo Fisher Scientific), then developed using a chemiluminescence imaging analysis system (ImageQuant LAS4000 Scanner).

### Statistical analysis

Data were analyzed using SPSS 19.0 (IBM, Armonk, NY USA). Continuous variables were reported as mean and standard deviation. Inter-group differences were assessed for significance using ANOVA, and multiple comparisons were performed using the Tukey method. Differences were considered significant if associated with a two-tailed *p* < 0.05.

### Ethics approval and Consent to participate

This study was approved by the Ethics Committee of West China Hospital. All procedures involving human participants were performed in accordance with the ethical standards of our institutions as well as the 1964 Helsinki Declaration and its later amendments. Patients or their families gave written informed consent for their tissues to be donated for this research.

### Consent for publication

Patients or their families gave written informed consent for the analyses of their tissues to be published.

## Results

### Patient characteristics

Characteristics of tissue donors and blood vessels are shown in Table 2. Tissues analyzed by human antibody array and Western blot came from four patients receiving a transplanted heart because of dilated cardiomyopathy, one patient receiving a transplanted heart because of coronary artery disease, and one patient who died in a traffic accident and donated his heart for heart transplantation, but the heart could not be used because of coronary atherosclerosis. Samples of other types of artery came mainly from cancer patients. Donors of heart tissue were younger than donors of other tissues.

**Table 2:** Characteristics of donors and their artery samples.

Characteristic	Type of artery (each n = 6)				
	Coronary	Mesenteric	Peripheral^a^	Pulmonary	Renal^b^
Age, yr	34 ± 8	56 ± 14	50 ± 14	51 ± 13	40 ± 10
Male	3 (50.0)	4 (66.7)	4 (66.7)	5 (62.5)	4 (66.7)
Diagnosis	Brain death (5); dilated cardiomyopathy (1)	Colorectal cancer (6)	Limb cancer (4); severe trauma (1); internal mammary artery (1)	Lung cancer (5); pulmonary hydatid disease (1)	Renal cortical atrophy (3); cancer (2); cyst (1)
Vascular outer diameter, mm	3.18 ± 0.81	2.63 ± 0.39	2.24 ± 0.63	3.36 ± 0.91	3.65 ± 0.40
Weight per unit area, g/mm^2^	0.34 ± 0.05	0.30 ± 0.05	0.29 ± 0.14	0.22 ± 0.05	0.35 ± 0.07

Values are n, n (%) or mean ± SD, unless otherwise noted.

^a^Includes two samples of posterior tibial artery and one sample each of dorsal artery, ulnar artery, popliteal artery, and internal mammary artery.

^b^Includes two patients with renal hypertension.

### Distribution of vasoactive receptors across five artery types

The relative expression levels of the 29 receptors was similar across renal, mesenteric, and peripheral artery types, with arginine vasopressin receptor 1A (AVPR1A) the most abundant receptor, followed by the ligand endothelin 1 (ET1), and then similarly abundant alpha2B-adrenoceptor (ADRA2B), alpha2C-adrenoceptor (ADRA2C), muscarinic cholinergic receptor (CHRM) and nicotinic cholinergic receptor (CHRNA).

In coronary artery, AVPR1A and ET1 were again the most abundant, followed by ADRA2B. CHRM and CHRNA were expressed at relatively low levels. Prostaglandin I2 receptor (PTGIR) was expressed at relatively high levels in coronary artery, but not significantly more than in other artery types (Supplementary Fig. 2).

In pulmonary artery, ET1 was expressed most abundantly, followed by AVPR1A and then by the four receptors ADRA2B, ADRA2C, AHRM and CHRNA, which were expressed at similar levels.

The angiotensin II receptor (AGTR), 5-hydroxytryptamine receptor (HTR), and thromboxane A2 receptor (TBXA2R) were expressed at low levels in all artery types (Supplementary Figs. 2 and 3a), while ADRB1, ADRB2 and ADRB3 were expressed at the lowest levels in all artery types (Supplementary Fig. 3b).

#### Alpha-adrenoceptors

**Figure 1:**
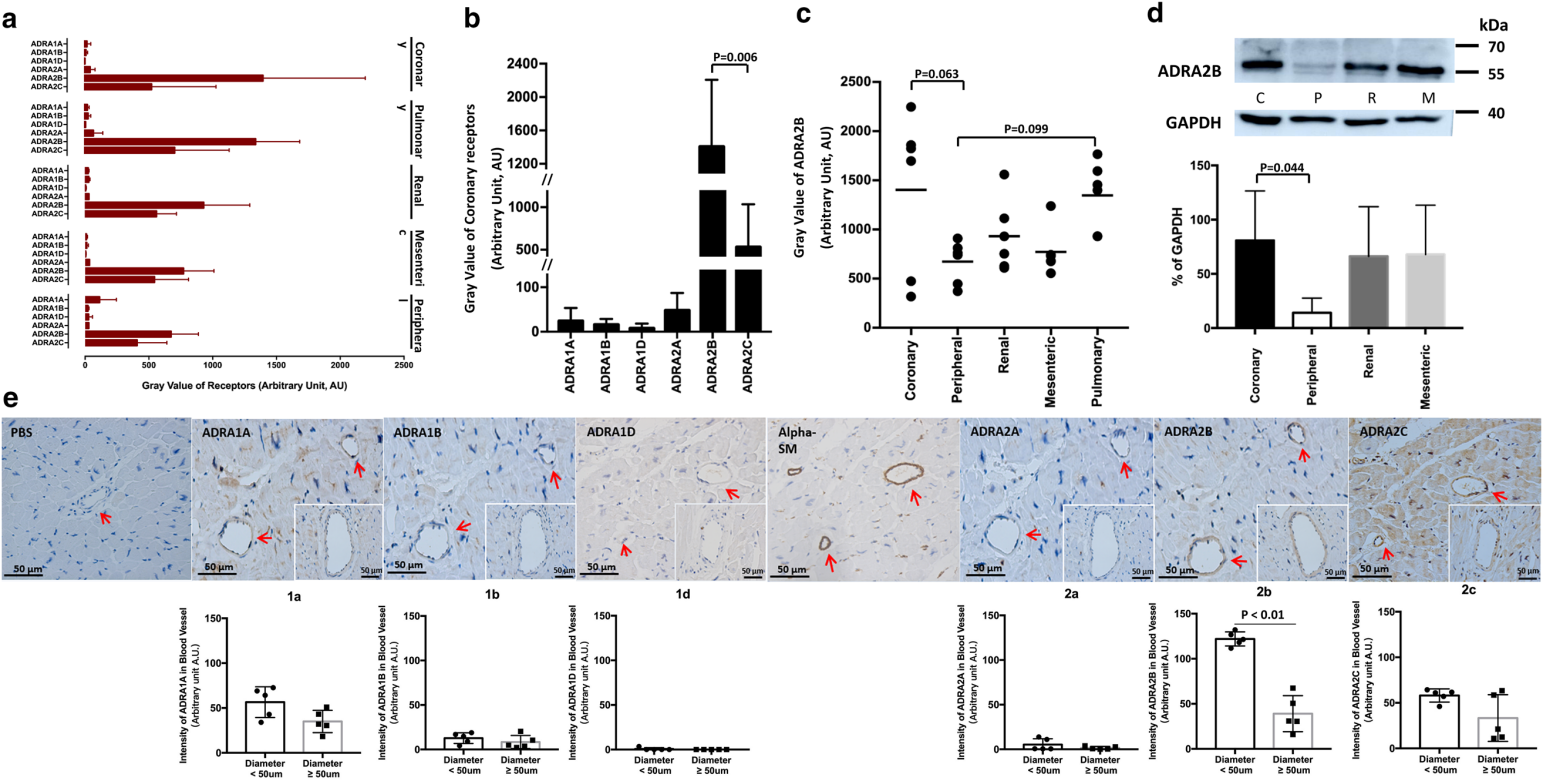
(**a**) Gray values of six subtypes of alpha-adrenergic receptor (ADRA) in coronary, pulmonary, renal, mesenteric and peripheral arteries. (**b**) Gray values of six subtypes of ADRA in coronary artery. (**c**) Gray values of ADRA2B in five types of arteries. (**d**) Western blot of ADRA2B in coronary (C), peripheral (P), renal (R), and mesenteric artery (M). GADPH served as a loading control. (**e**) Immunohistochemistry against six subtypes of ADRA in myocardium. Sections were stained against alpha smooth muscle actin (alpha-SM) to locate arteries. Red arrows: artery with diameter < 50 μm. Bottom right corner: artery with diameter ≥ 50 μm. As a negative control, sections were incubated with phosphate-buffered saline instead of primary antibody. Data are mean ± SD, and P < 0.05 for Student’s *t* test was set as the significance threshold.

In all types of artery, ADRA2B and ADRA2C were expressed at higher levels than ADRA1A, ADRA1B, ADRA1D or ADRA2A (Fig. 1a). In renal, mesenteric and peripheral arteries, ADRA2B and ADRA2C were expressed at similarly high levels, but ADRA2B in coronary artery (gray value 1403 ± 802) was expressed at a significantly higher level than ADRA2C (530 ± 505, *p* = 0.006; Fig. 1b) and a slightly higher level than ADRA2B in peripheral artery (634 ± 214, *p* = 0.063; Fig. 1c). These results were confirmed by Western blot (Fig. 1d) and immunohistochemistry (Fig. 1e). The latter technique further showed that ADRA2C was expressed in both coronary artery and myocardium, and that ADRA2B expression was higher in arteries with diameters smaller than 50 μm than in larger arteries (Fig. 1e).

ADRA1A and ADRA1B were observed in myocardium at lower levels than ADRA2C, but they were barely detectable in arteries. ADRA1D and ADRA2A were scarcely expressed in either myocardium or blood vessels.

#### Arginine vasopressin receptors and endothelin

**Figure 2:**
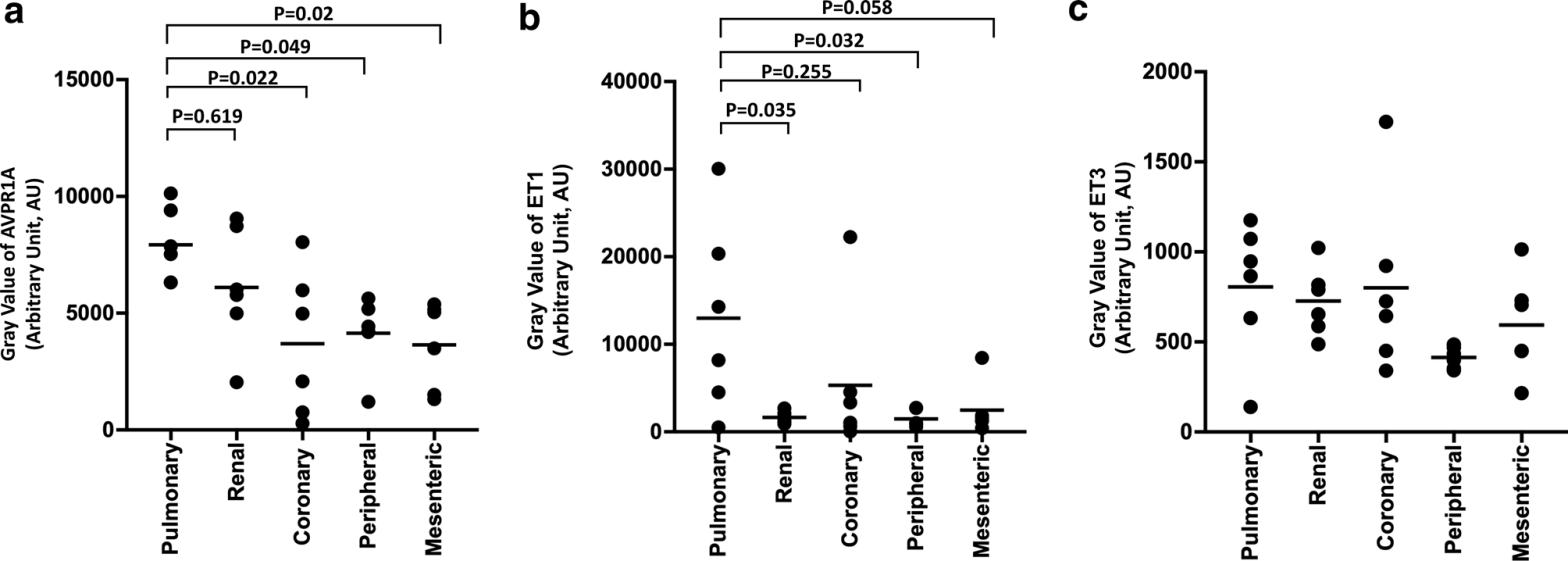
Gray values of (**a**) arginine vasopressin receptor 1A (AVPR1A), (**b**) endothelin 1 (ET1), and (**c**) endothelin 3 (ET3) in five types of arteries. Data are mean ± SD.

Among the 32 detected receptors and ligands, AVPR1A showed the highest expression levels across all five artery types (mean gray value 5102 ± 1873). The expression level of AVPR1A in pulmonary artery (7931 ± 1574) was similar to that in renal artery (6103 ± 2586), 2.2 times that in mesenteric artery (3648 ± 1857, *p* = 0.02), 1.9 times that in peripheral artery (4143 ± 1550, *p* = 0.049), and 2.2 times that in coronary artery (3690 ± 3119, *p* = 0.022; Fig. 2a).

ET1 was highly expressed across all types of arteries. The level in pulmonary artery (mean gray value 12,982 ± 10,926) was 8.8 times that in peripheral artery (1476 ± 976, *p* = 0.032), 5.3 times that in mesenteric artery (2475 ± 2964, *p* = 0.058), 7.9 times that in renal artery (1638 ± 668, *p* = 0.035), and 2.4 times that in coronary artery (5319 ± 8479, *p* = 0.255) (Fig. 2b). ET3 was expressed at similar levels across all types of arteries (Fig. 2c), which were lower than the levels of ET1. ET2 was barely detectable across all types of arteries (Supplementary Fig. 4a).

AVPR1B and AVPR2 were expressed at much lower levels than AVPR1A (Supplementary Fig. 4b).

#### Cholinergic and other receptors

**Figure 3:**
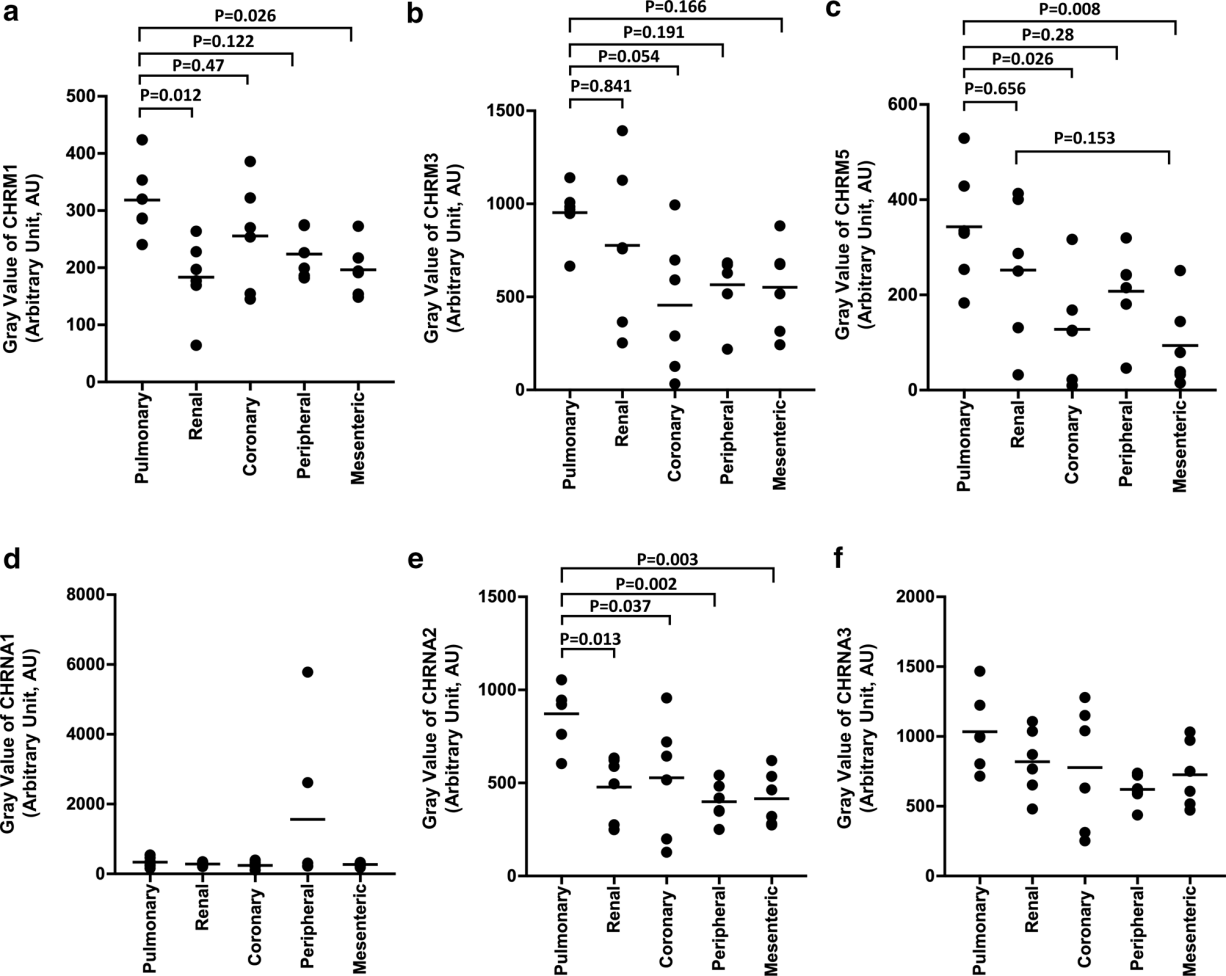
Gray values of various cholinergic receptor subtypes in five types of arteries: (**a**) muscarinic 1 (CHRM1), (**b**) muscarinic 3 (CHRM3), (**c**) muscarinic 5 (CHRM5), (**d**) nicotinic alpha 1 (CHRNA1), (**e**) nicotinic alpha 2 (CHRNA2), and (**f**) nicotinic alpha 3 (CHRNA3). Data are mean ± SD.

CHRM3 was the most abundantly expressed muscarinic receptor and its level in pulmonary artery (mean gray value 953 ± 156) was 1.7 times the level in peripheral artery (565 ± 180, *p* = 0.191), 1.7 times that in mesenteric artery (552 ± 241, *p* = 0.166), 1.2 times that in renal artery (777 ± 435, *p* = 0.841, and 2.1 times that in coronary artery (456 ± 369, *p* = 0.054; Fig. 3b). The expression of CHRM1, CHRM3 or CHRM5 did not vary substantially with artery type (Fig. 3a-c), and CHRM2 was barely detectable in all artery types (Supplementary Fig. 5a).

Expression of CHRNA1 and CHRNA 3 (Fig. 3d,f) as well as CHRNA4 and CHRNA5 (Supplementary Fig. 5b) did not vary substantially with artery type. CHRNA2 expression in pulmonary artery (mean gray value: 872 ± 161) was 1.8 times that in renal artery (478 ± 174, *p* = 0.013), 1.7 times that in coronary artery (528 ± 317, *p* = 0.037), 2.2 times that in peripheral artery (399 ± 105, *p* = 0.002), and 2.1 times that in mesenteric artery (416 ± 145, *p* = 0.003) (Fig. 3e).

## Discussion

Various mediators and their receptors regulate the balance between vasoconstriction and vasodilation, and perturbation of this equilibrium can cause life-threatening disease. Here we provide what appears to be the first description of the distribution of 29 vasoactive receptors and 3 ligands in various types of human arteries. Our results suggest that AVPR1A is expressed at higher levels than any receptor that we examined, regardless of the artery type, and that it is expressed at lower levels in coronary artery than in renal, pulmonary, mesenteric or systemic arteries. ADRA2B was expressed at higher levels in coronary artery than other types of alpha-adrenoceptors, while ET1 was expressed at higher levels in pulmonary artery. These insights into tissue-specific distribution of vasoactive receptors and ligands may help guide the clinical use of certain vasoactive agents that limit drug activity to specific artery types.

We focused on receptors in the sympathetic nerve system because of their potential association with the development of coronary artery atherosclerosis and acute myocardial ischemia. These receptors can activate platelets and thereby promote atherosclerosis, and they can constrict the coronary artery, triggering acute ischemic events^16^. Sudden activation of sympathetic nerves, such as during severe emotional disturbance, can induce acute cardiovascular events in individuals with coronary artery atherosclerosis^17^. This surge in sympathetic nerve activity can increase the myocardial infarction area^18^ and lead to poor prognosis after ischemia^19,20^, including sudden cardiac death. Blocking the thoracic sympathetic preganglionic fibers inhibits release of sympathetic neurotransmitters acting on coronary arteries, which can substantially increase coronary blood flow^21^. Similarly, high epidural block can relieve angina and improve the prognosis of patients with coronary heart disease^22^.

Our results identify ADRA2B as a novel therapeutic target in ischemic heart disease. This receptor was expressed more strongly in coronary artery, especially in vessels with diameters below 50 μm, than in other artery types. Specific agonists of this and other alpha 2-adrenorceptors significantly constrict the coronary artery and reduce coronary blood flow^23,24^. The D/D genotype of the *ADRA2B* gene leads to a receptor that hyper-responds to adrenaline, decreasing coronary blood flow^25^ and increasing risk of acute myocardial infarction and sudden cardiac death^26–28^.

We found the most abundant vasoactive receptors in myocardium to be ADRA1A, ADRA2A, and ADRA2C. It is possible that during stimulation of the sympathetic nervous system, these receptors are activated by norepinephrine to increase contractility. This may, in turn, increase oxygen consumption and lead to myocardial ischemia.

In our samples of systemic artery, AVPR1A was more strongly expressed than any other assayed receptor. Interestingly, blood pressure is sensitive to arginine vasopressin^29^. The high expression of AVPR1A may contribute to the sensitive response of blood pressure to arginine vasopressin. Using vasopressin to treat hypotension can induce mesenteric and digital ischemia^30–32^ as well as skin necrosis^33^, and vasopressin treatment is associated with higher risk of digital ischemia than norepinephrine treatment^34^. Whether these side effects are associated with high expression of AVPR1A need further functional studies.

AVPR1A was expressed at a moderate level in coronary artery. This supports the use of low-dose arginine vasopressin to treat hypotension while minimizing interference with the coronary artery. High vasopressin concentration leads to coronary vasoconstriction and myocardial depression in isolated animal hearts^35,36^, and the AVPR1A agonist selepressin increases coronary vascular resistance in a canine model^37^. High-dose arginine vasopressin infusion significantly reduces left ventricular ejection fraction and increases mortality in a mouse model of ischemia/reperfusion^38^. Delivering vasopressin to patients at < 0.03 U/min improves cardiac output^34,39^ and reduces risk of renal failure and mortality^40^, but delivering it at > 0.05 U/min increases risk of cardiac arrest^39^ and other poor outcomes^41^.

We found that ET1 was abundant in pulmonary artery, consistent with its potent vasoconstrictive activity^42^ and its involvement in pulmonary arterial hypertension^43^. Indeed, the ET receptor antagonist bosentan can significantly improve hemodynamics, reduce severity of pulmonary hypertension and strengthen motor ability of patients with pulmonary hypertension^44,45^. The ET receptor antagonists maxitetan and ambesantan can also increase arterial oxygen saturation and the exercise ability of patients with pulmonary hypertension^46,47^. Our results suggest that another strategy to treat pulmonary hypertension may be to block ET1 synthesis or its activity using a specific antagonist.

In our study, pulmonary artery expressed abundant cholinergic receptors, where they regulate not only vasodilation and vasoconstriction, but also inflammation, airway remodeling, and airway mucus secretion. Indeed, activation of CHRM3 has been implicated in pulmonary artery contraction^48^. Muscarinic receptor antagonists are widely used to treat asthma^49^, chronic obstructive pulmonary disorder^50^ and pulmonary hypertension^51^. The acetylcholinesterase inhibitor pyridostigmine is used to reduce pulmonary vascular resistance and remodeling, as well as right ventricle afterload^51^. Our results showed that CHRM3 has a relatively abundant expression compared to other muscarinic receptors across all type of arteries. This may suggest that CHRM3 plays important physiological roles in various arteries. Future studies should examine whether CHRM3 agonists or antagonists can be used to treat disorders of other organs.

Our results should be interpreted carefully because of several limitations. First, tissues came from older and younger patients, and receptor expression patterns may differ with age. Second, it is not always safe to assume that the relative abundance of a given receptor reflects its vasoconstrictive effects. For example, AVPR1A is highly expressed in systemic and pulmonary arteries, but it induces vasoconstriction only in the former^52^. Deoxyepinephrine induces vasoconstriction 10 times more strongly in systemic than pulmonary artery, suggesting that alpha-adrenoceptors may respond differently depending on the tissue. Third, receptors can behave differently under normal or pathological conditions. For example, ADRA2 triggers vasospasm and contraction of coronary artery in patients with coronary atherosclerosis but not in individuals with normal coronary artery^24^. Such differences may reflect differences in available levels of receptor ligand. For example, patients with hypertension or symptomatic atherosclerosis have elevated plasma levels of ET1^53^. Therefore, the distribution and effects of receptors in various vascular diseases need further study. Fourth, we found that patients varied substantially in expression of certain receptors in coronary artery (e.g. AVPR1A, ET1 and PTGIR). While this variability may make our results less generalizable, it implies that these receptors may be targets for precision medicine. Fifth, we were unable to sample all types of arteries because some types, such as cerebral artery, are difficult to sample; moreover, our protein array did not contain certain vasoactive substances synthesized by endothelial cells (e.g. NO, PGI2 and TXA2) because they are unstable. Lastly, we did not analyze receptor functionality in our study, which should be the subject of future work.

Despite these limitations, our results may provide an important reference for developing tissue-specific vasoactive drugs against hypotension, ischemic heart disease, and pulmonary hypertension.

## Supplementary information

Supplementary figures.

## Data Availability

The original data on which this work is based are available from the corresponding author on reasonable request, depending on national laws and international regulations regarding sharing of confidential patient data.
