# Supplementary material for: Distribution and relative expression of vasoactive receptors on arteries
Source: Sci Rep. 2020 Sep 21;10:15383. doi: 10.1038/s41598-020-72352-5 (PMC7505843; doi:10.1038/s41598-020-72352-5)
Supplement: Supplementary file 1 — Supplementary figures. [file 41598_2020_72352_MOESM1_ESM.docx]

**Distribution and relative expression of vasoactive receptors on arteries**

Xinhao Liu^1#^, Dan Luo ^1#^, Jie Zhang ^2^, Lei Du ^1^*

^1^ Department of Anesthesiology and Translational Neuroscience Center, West China Hospital, Sichuan University

^2^ Key Laboratory of Transplant Engineering and Immunology, West China Hospital, Sichuan University

**Supplementary Figure 1.**

**
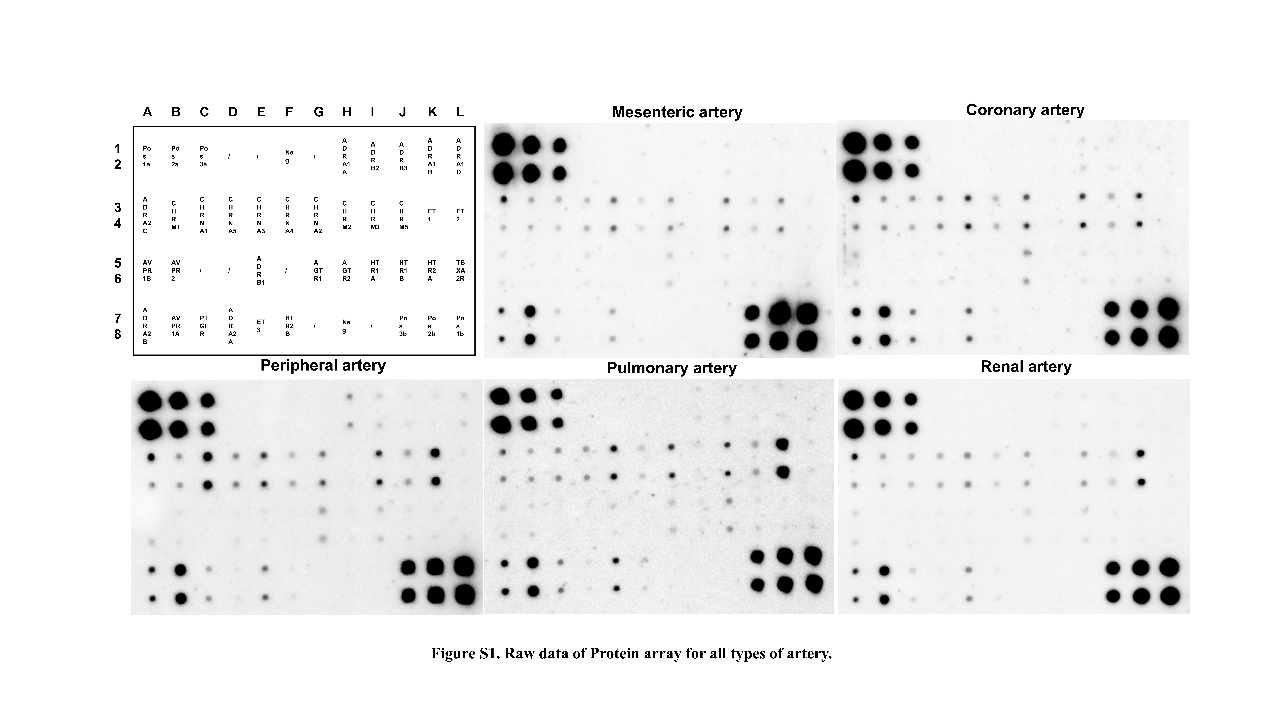
**

**Supplementary Figure 1.** Array of human antibodies against vasoactive receptors and representative results for five types of arteries. Pos1a, Pos2a, Pos3a and Pos1b, Pos2b, Pos3b were different concentrations of biotinylated bovine serum albumin spotted onto the array to serve as positive controls and to allow cross-array normalization. “Neg” indicates negative control spots containing phosphate-buffered saline. “/” indicates blank spots.

**Supplementary Figure 2**

**
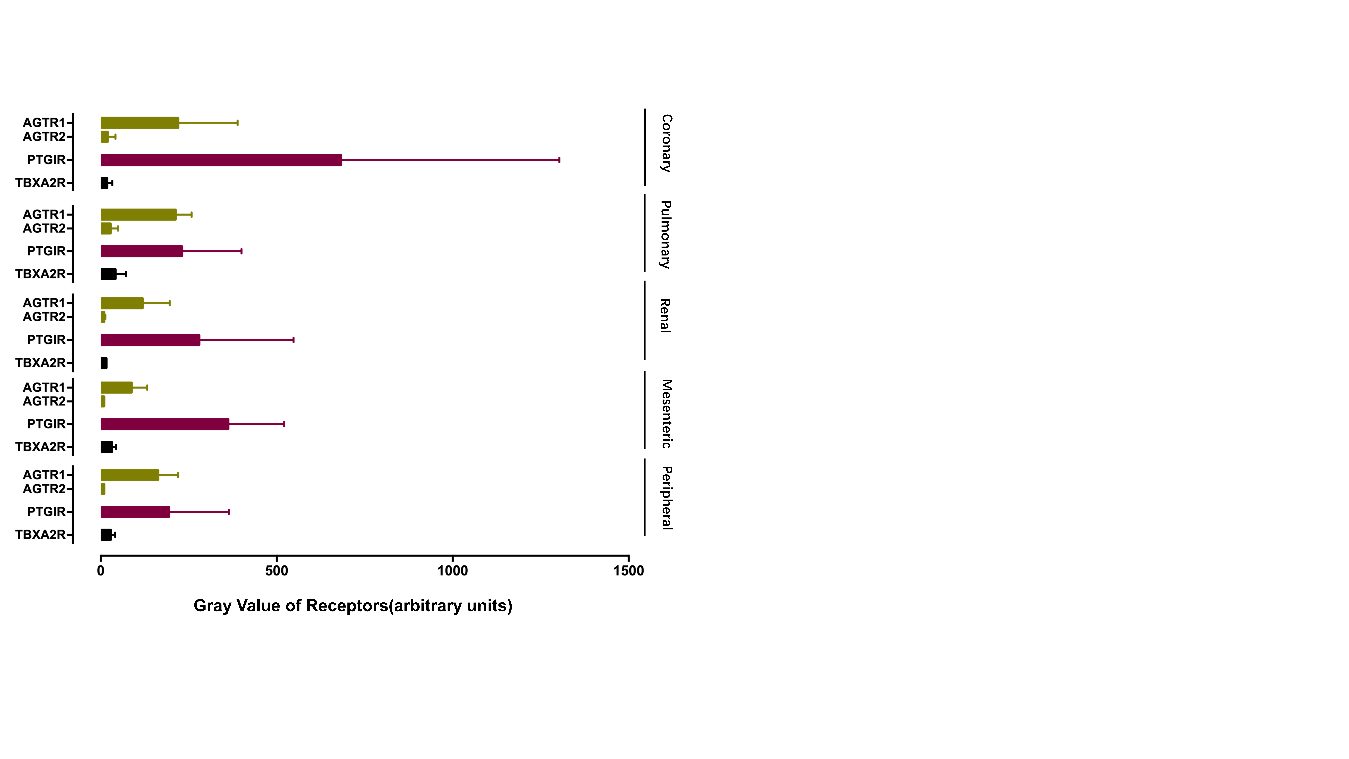
**

**Supplementary Figure 2.** Gray values of arginine vasopressin receptor (AGTR), prostaglandin I2 receptor (PTGIR) and thromboxane A2 receptor (TBXA2R) in five types of artery. data were presented as mean ± SD

**Supplementary Figure 3**

**
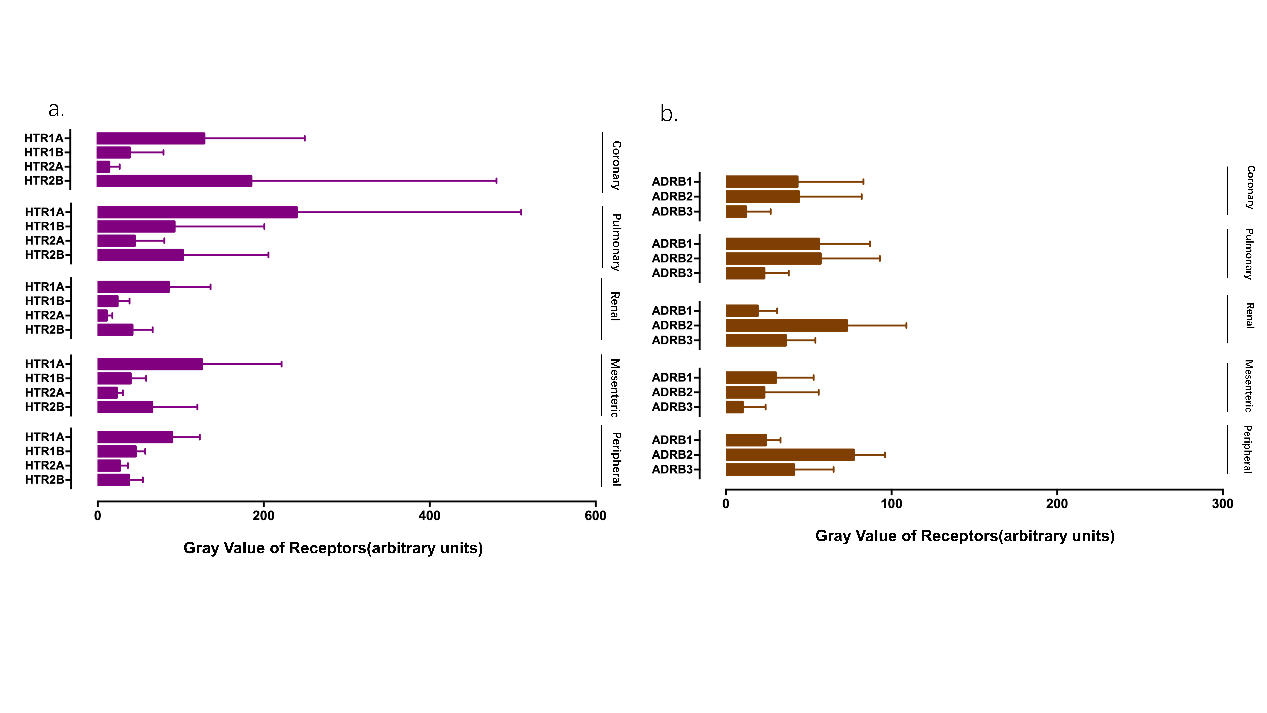
**

**Supplementary Figure 3.** Gray values of (a) 5-hydroxytryptamine receptor (HTR) subtypes and (b) beta-adrenergic receptor (ADRB) subtypes in five types of artery. data were presented as mean ± SD

**Supplementary Figure 4**

**
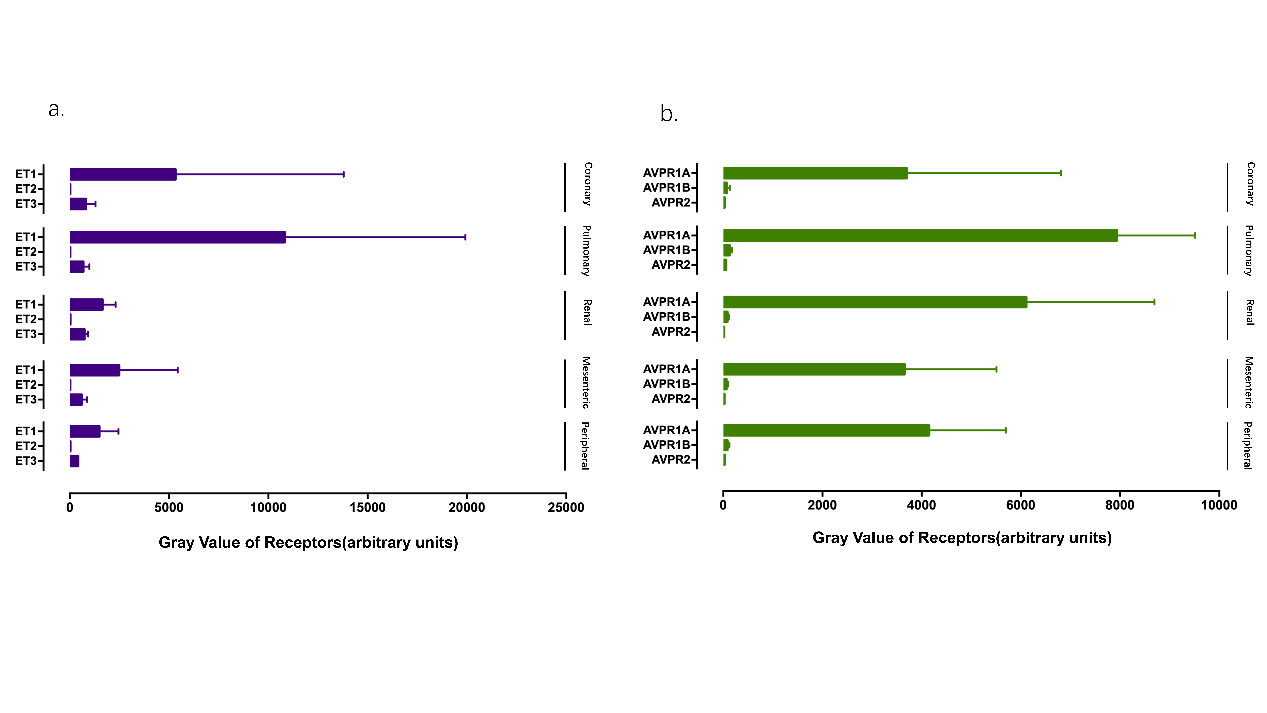
**

**Supplementary Figure 4.** Gray values of (a) endothelin (ET) subtypes and (b) arginine vasopressin receptor (AVPR) subtypes in five types of artery. data were presented as mean ± SD

**Supplementary Figure 5**

**
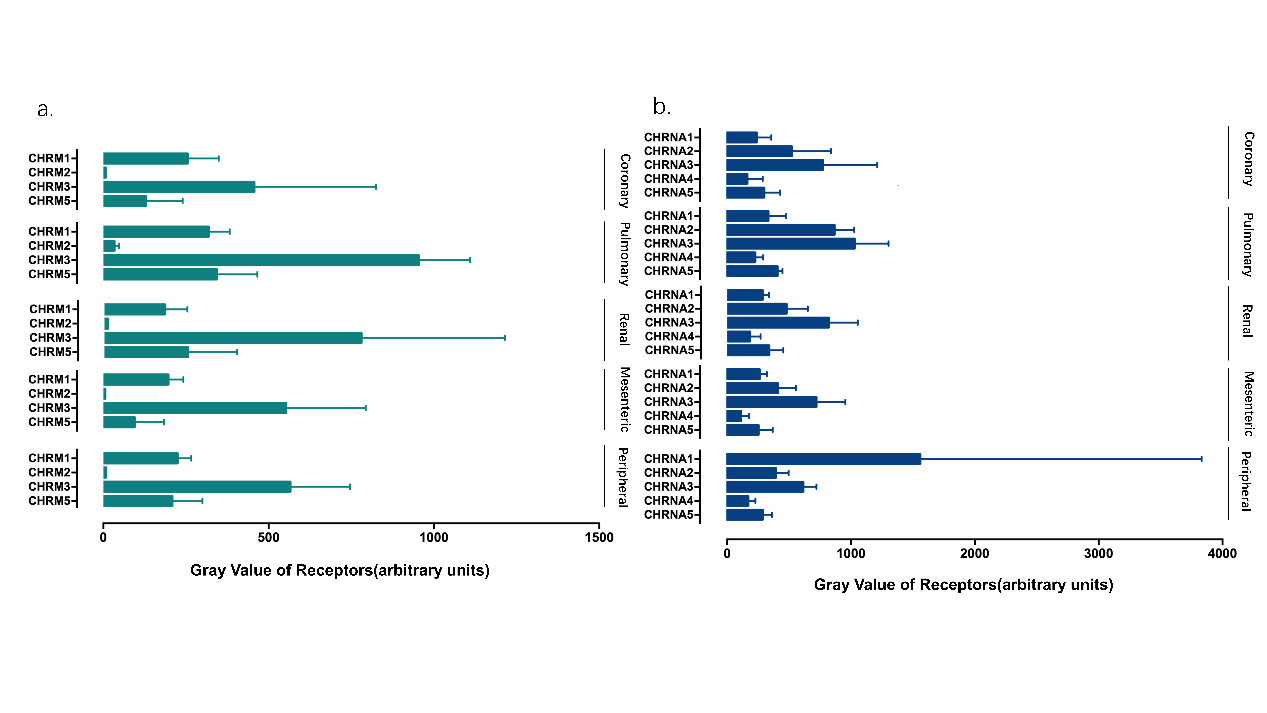
**

**Supplementary Figure 5.** Gray values of (a) cholinergic muscarinic receptor (CHRM) subtypes and (b) cholinergic nicotinic receptor (CHRNA) subtypes in five types of artery. data were presented as mean ± SD

**Supplementary 6.**


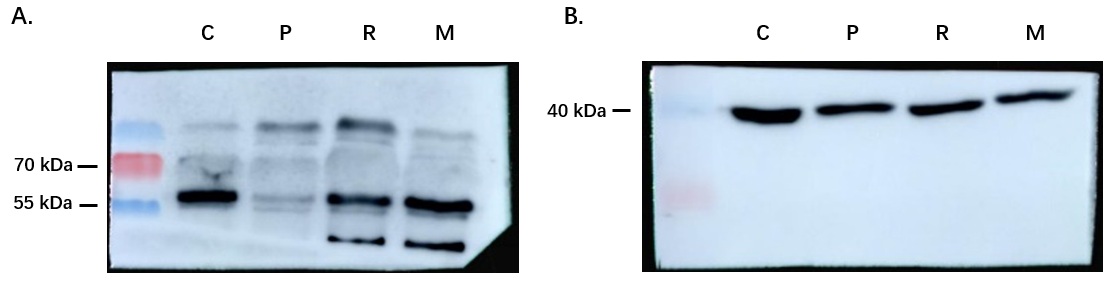


**Supplementary 6. Full image of Western blot of ADRA2B and GAPDH in Figure 1d.** (A) Western blot of ADRA2B in coronary (C), peripheral (P), renal (R), and mesenteric artery (M). (B). Western blot of GADPH in coronary (C), peripheral (P), renal (R), and mesenteric artery (M).
